# Targeting the N-Terminus Domain of the Coronavirus Nucleocapsid Protein Induces Abnormal Oligomerization via Allosteric Modulation

**DOI:** 10.3389/fmolb.2022.871499

**Published:** 2022-04-19

**Authors:** Jia-Ning Hsu, Jyun-Siao Chen, Shan-Meng Lin, Jhen-Yi Hong, Yi-Jheng Chen, U-Ser Jeng, Shun-Yuan Luo, Ming-Hon Hou

**Affiliations:** ^1^ Institute of Genomics and Bioinformatics and Department of Life Sciences, National Chung Hsing University, Taichung, Taiwan; ^2^ Department of Chemistry, National Chung Hsing University, Taichung, Taiwan; ^3^ National Synchrotron Radiation Research Center, Department of Chemical Engineering, National Tsing Hua University, Hsinchu, Taiwan

**Keywords:** PPI-based drug design, n protein, allosteric modulator, COVID-19, MERS-CoV

## Abstract

Epidemics caused by coronaviruses (CoVs), namely the severe acute respiratory syndrome (SARS) (2003), Middle East respiratory syndrome (MERS) (2012), and coronavirus disease 2019 (COVID-19) (2019), have triggered a global public health emergency. Drug development against CoVs is inherently arduous. The nucleocapsid (N) protein forms an oligomer and facilitates binding with the viral RNA genome, which is critical in the life cycle of the virus. In the current study, we found a potential allosteric site (Site 1) using PARS, an online allosteric site predictor, in the CoV N-N-terminal RNA-binding domain (NTD) to modulate the N protein conformation. We identified 5-hydroxyindole as the lead via molecular docking to target Site 1. We designed and synthesized four 5-hydroxyindole derivatives, named P4-1 to P4-4, based on the pose of 5-hydroxyindole in the docking model complex. Small-angle X-ray scattering (SAXS) data indicate that two 5-hydroxyindole compounds with higher hydrophobic R-groups mediate the binding between N-NTD and N-C-terminal dimerization domain (CTD) and elicit high-order oligomerization of the whole N protein. Furthermore, the crystal structures suggested that these two compounds act on this novel cavity and create a flat surface with higher hydrophobicity, which may mediate the interaction between N-NTD and N-CTD. Taken together, we discovered an allosteric binding pocket targeting small molecules that induces abnormal aggregation of the CoV N protein. These novel concepts will facilitate protein-protein interaction (PPI)-based drug design against various CoVs.

## 1 Introduction

Recently emerging infections caused by coronaviruses (CoVs), including the severe acute respiratory syndrome (SARS), Middle East respiratory syndrome (MERS), and coronavirus disease 2019 (COVID-19), have led to a global public health emergency (Du Z. et al., 2020; Hui et al., 2020; Xu et al., 2020; Zhu et al., 2020). Interestingly, all the aforementioned CoVs belong to the β subgroup of the *Coronaviridae* family, which are characteristically single-stranded RNA viruses capable of circulating among mammals, such as bats, civet cats, and camels (Hui et al., 2020) and can cause respiratory illness in humans during epizootic spillovers. Therefore, the development of effective antiviral drugs and vaccines against these CoV infections is of the utmost importance to prevent their further spread (Ghosh et al., 2020; Khodadadi et al., 2020). The CoV genome contains four structural proteins: nucleocapsid (N), small envelope (E), matrix (M), and trimeric spike (S) glycoproteins, which are essential for virion assembly and function to complete the viral life cycle during infections. Among the structural proteins of CoVs, N proteins form a major structural component, are relatively evolutionarily conserved, and share the same modular organization (Chang et al., 2014) which consists of intrinsically disordered regions (IDRs): N-arm, C-arm, and two structural domains, including the N-terminal RNA-binding domain (NTD) and C-terminal dimerization domain (CTD) (Wootton et al., 2002; Chang et al., 2014). Dimeric N protein functions as a building block by binding to the viral RNA, forming a ribonucleoprotein (RNP) complex, a primary part of viral self-assembly, where subsequent viral replication and translation can proceed (Almazan et al., 2004; Chang et al., 2005; Jayaram et al., 2006; Zuniga et al., 2010). Furthermore, the N protein is also involved in regulating the host cell cycle and viral pathogenesis, ultimately facilitating production of the virus (McBride et al., 2014). T These multifunctional characteristics of the N protein and its low rate of mutation make it a prominent target for the development of therapeutics against CoVs (Lin et al., 2014; Mori et al., 2015; Hu et al., 2017).

Two strategies can be used to inhibit the function of N proteins against CoVs. The first strategy is to develop antiviral agents that target the RNA-binding site of the N protein and specifically block RNP formation during viral replication. The second strategy is to affect the normal N protein oligomerization by inducing or inhibiting protein-protein interactions (PPIs) between N protein molecules. Modulation of PPIs can be achieved by the development of compounds that either bind to the PPI interaction surface, which directly affects the associated PPI (orthosteric modulators) or bind to the adjacent site of the PPI interface (allosteric modulators), which induces a conformational change to inhibit or enhance the PPIs of the target protein complexes (Thiel et al., 2012; Fischer et al., 2015; Modell et al., 2016; Petta et al., 2016). In general, allosteric modulators have the potential for greater subtype selectivity and do not need to compete with the bulky and relatively high-affinity PPI partners when compared to orthosteric ligands. Moreover, allosteric modulators possess no intrinsic activity of their own, and thus, the side effects for clinical use could be reduced.

Most CoV N-NTD structures are folded in a monomeric conformation. In contrast, the CoV N-CTDs are always dimeric and responsible for N protein oligomerization via PPIs. In this study, we discovered one potential site, Site 1, in the MERS-CoV N-NTD with an online software, PARS, developed based on the normal-mode analysis method for designing an allosteric modulator. We utilized molecular docking technology to identify 5-hydroxyindole from an affordable 96-compound library designed by Muelle’s group as the potential lead by targeting Site 1. We designed and synthesized four 5-hydroxyindole derivatives, named P4-1 to P4-4, based on the pose of 5-hydroxyindole in the docking model complex. Two 5-hydroxyindole derivatives, containing higher hydrophobic R-groups, exhibited the ability to elicit the oligomerization of N protein by small-angle X-ray scattering (SAXS) experiments. We also described the structure of MERS-CoV N-NTD complexed with P4 series compounds and revealed that higher hydrophobic R-group derivatives contribute to the hydrophobicity through the creation of flat interfaces between N-NTD and N-CTD. This unique approach based on targeting N-NTD to change the N protein structure by allosteric modulators can be potentially applied to discover novel drugs against CoV diseases, including SARS-CoV-2.

## 2 Results

### 2.1 Discovery of the Protein Pocket of N-NTD for Drug Design

**FIGURE 1:**
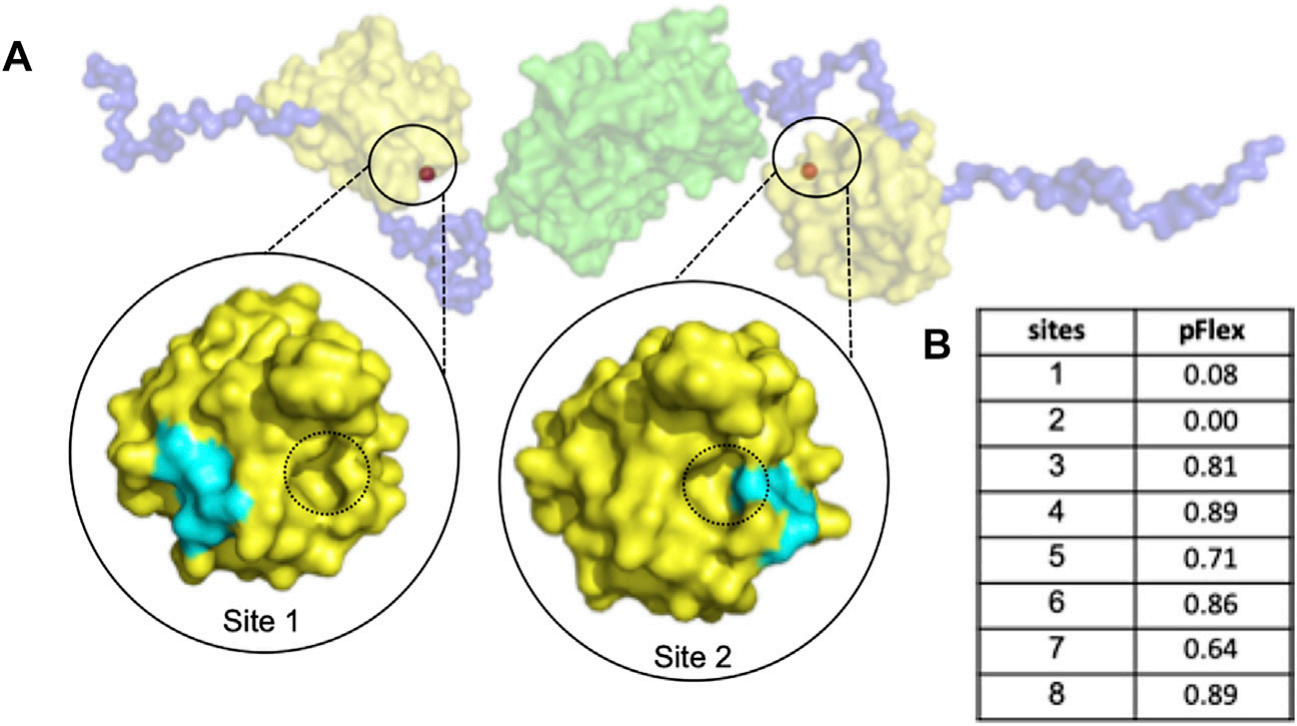
The dimeric MERS-CoV N protein possesses a druggable sites for allosteric modulator design. **(A)** The structure of dimeric N protein was obtained by SAXS experiment from previous publication (Lin et al., 2020) in which the NTD, linker and dimeric CTD were shown in yellow, blue and green, respectively. The predicted allosteric sites were shown in orange and highlighted in black circles. For simplification, only the sites with potent pFlex were shown. The interface of non-native PPI was indicated with black dotted circles. **(B)** The pFlex values of all predicted sites. pFlex indicates overall flexibility of target protein may be affected by the binding ligand to the sites where the *p*-value is lower than 0.05.

The abnormal aggregation of the N protein is induced by small molecules which manipulate a non-native PPI interaction that inhibits CoV replication (Lin et al., 2020); Thus, we proposed identifying a suitable cavity for the CoV N-NTD to induce abnormal N protein aggregation via allosteric modulation. To this end, we detected the pockets suitable for drug design using PARS, an online tool for allosteric site prediction based on the normal-mode analysis method (Panjkovich and Daura, 2014), with the dimeric structure of the N protein solved by SAXS experiments. Two sites on the protein surface were identified to have the potential for allosteric modulator design (Sites 1 and 2). Since site 2 is too close to the interface of non-native PPI, it may interfere with allosteric modulation; therefore, we selected site 1 for further design (Figure 1).

Docking studies were performed on the selected cavity using the Discovery Studio Client (v20.1.0.18287) with an assembled fragment library designed by Huschmann et al., characterized by a broad ligand diversity and high hit rate to the protein surface (Huschmann et al., 2016), to determine the starting chemical lead with high docking scores. Because low-molecular-weight ligands are considered to have the advantage of facilitating their optimization into potent compounds with drug-like properties (Murray and Rees, 2009), we excluded larger compounds. Thirty-two different structures with molecular weights (MWs) ranging from 150 to 250 Da were shortlisted. Second, the Pi-stacking interaction is commonly observed in protein-ligand complexes (Ferreira de Freitas and Schapira, 2017); therefore, we excluded ligands that lacked aromatic rings in their structures. Third, the hydrophobic interaction plays a key role in protein aggregation; therefore, we excluded the hydrophilic fragments. Following the aforementioned criteria to exclude ligands, we narrowed down the potential ligands to the four fragments listed in Table 1. Among them, the 5-hydroxyindole scaffold stood out owing to its diverse biological activities and potential use in the medical industry (Kochanowska-Karamyan and Hamann, 2010; Ishikura et al., 2015). Furthermore, it has been used as a lead compound to develop antiviral agents, such as bufotenine (Vigerelli et al., 2020) and the antiviral drug for influenza, arbidol (Herod et al., 2019; Du F. et al., 2020). Therefore, we chose 5-hydroxyindole (P4) for further experiments.

**TABLE 1:** Detailed information of the final fragments after screening procedures.

Molecule	Name	MW [Da]	tPSA	LigScose	logP (o/w)	Main Function
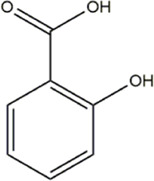	Salicylic acid	138.12 g/mol	57.53	50.8536	1.27	Anti-inflammation
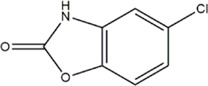	chlorzoxazone	169.56 g/mol	38.33	57.729	1.8	Skeletal muscle relaxants
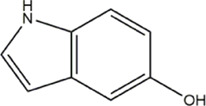	5-Hydroxyindole	133.15 g/mol	36.02	52.7897	2	Anti-virus
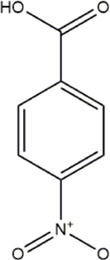	4-Nitrobenzoic acid	167.12 g/mol	83,12	50.4913	1.52	N/A

### 2.2 Design and Synthesis of 5-Hydroxyindole Derivatives by Targeting the Potential Allosteric Site of CoV N-NTD

**FIGURE 2:**
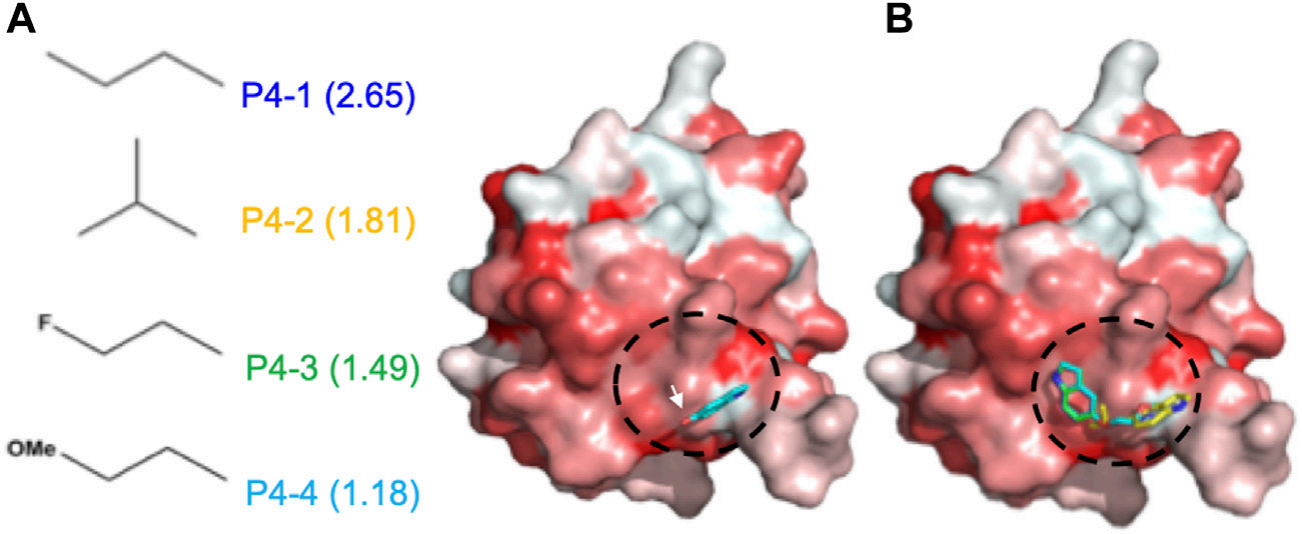
Docking results of CoV N-NTD with 5-Hydroxyindole. **(A)** (left) The structures of each chemical moiety designed for 5-Hydroxyindole modification. The calculated miLogP values of each moiety were shown in brackets. (right) Surface representation of CoV N-NTD with the expected binding site of 5-Hydroxyindole, obtained by using the molecular docking. The surface was colored according to the hydrophobicity level at the protein surface. The chemical moieties were designed to add to the hydroxyl group of 5-Hydroxyindole (indicated by white arrow) to increase the hydrophobic contacts between each compound and the expected binding surface (indicated by black cycle) **(B)** Same as **(A)** except the 5-Hydroxyindole was replaced by P4-1, P4-2, P4-3, and P4-4 are shown in blue, yellow, green and cyan, respectively.

**Scheme 1:**
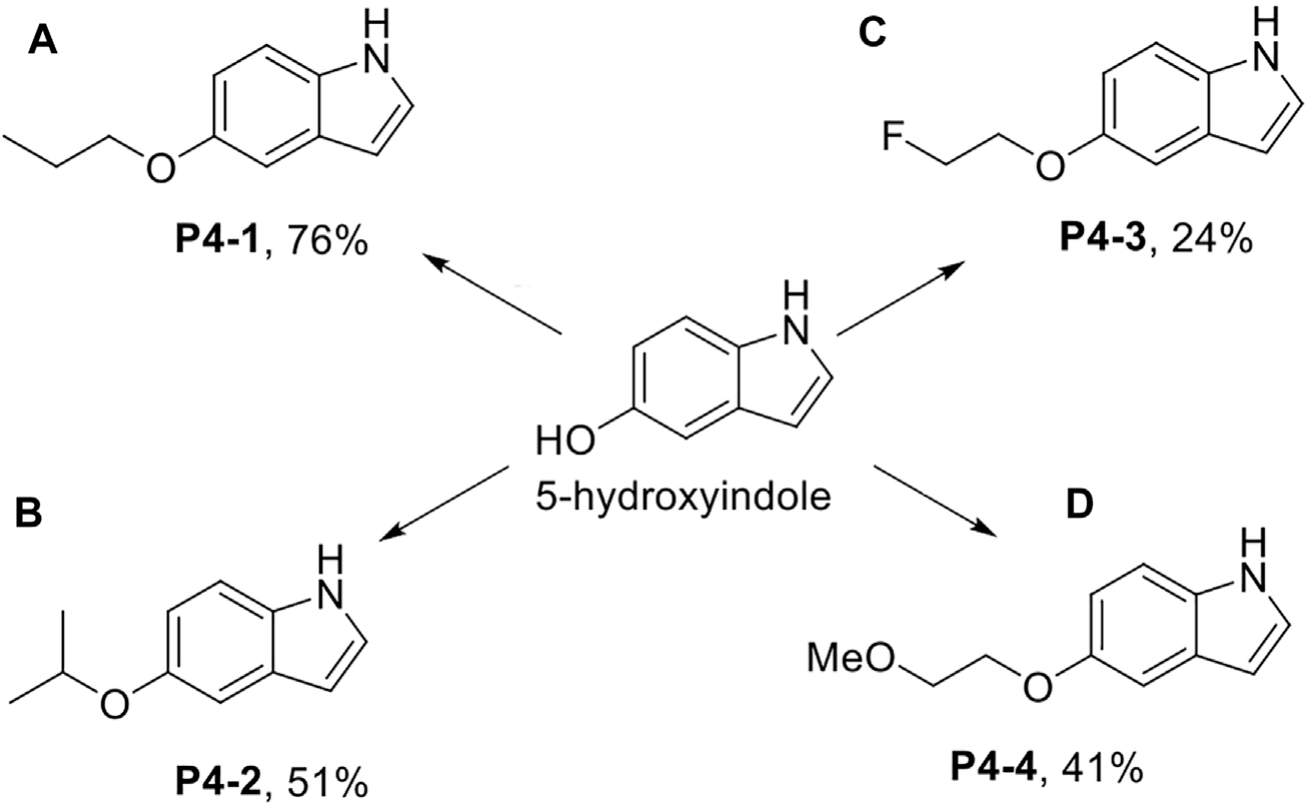
Synthesis of P4-1 to P4-4. Reagent and conditions: **(A)** NaH, DMF, 25°C, 30 min; then 1-iodopropane, 25°C, 3 h; **(B)** 2-bromopropane, NaH, DMF, 60°C, 6 h then 2-bromopropane, NaH, 60°C, 3h; **(C)** 1-bromo-2-fluoroethane, K_2_CO_3_, acetone, reflux, 12 h; **(D)** 1-iodo-2-methoxyethane, NaH, DMF, 25°C, 3 h.

Based on the pose of 5-hydroxyindole in the docking complex model (Figure 2A), we designed four derivative compounds with the modifications on the hydroxyl group at position C5, by which the modifications can extend to the predicted surface with higher binding affinity for allosteric modulation (Scheme 1). Furthermore, because hydrophobic interactions play an important role in the binding of allosteric modulators to their target proteins (Duan et al., 2019), we designed side chains with different degrees of hydrophobicity to strengthen these hydrophobic interactions. We named these four compounds as P4-1 to P4-4 and evaluated their abilities to modulate the protein oligomeric status. Molecular docking was performed again to evaluate the capacity of each compound to target the potent allosteric site. The results revealed that the P4-derived compounds fit well with the predicted surface (Figure 2B).

P4-1 to P4-4 were chemically synthesized. As shown in Scheme 1, we used 5-hydroxyindole and sodium hydride (NaH) with DMF as the solvent for deprotonation of the hydroxyl group and proceeded with 1-iodopropane to obtain P4-1 at a yield rate of up to 76% (Scheme 1A). Similar to P4-1 production, P4-2 was obtained, followed by the reaction with 2-bromopropane at a yield rate of 51% (Scheme 1B). To generate P4-3, 5-hydroxyindole was reacted with 1-bromo-2-fluoroethane and potassium carbonate (K_2_CO_3_) in acetone at a yield rate of 24% (Scheme 1C). As for P4-4, we referred to the procedures documented previously (Batista et al., 2008); 5-hydroxyindole was reacted with 1-iodo-2-methoxyethane and NaH in DMF to obtain P4-4 at a yield rate of 41% (Scheme 1D).

### 2.3 P4 Compounds Containing Hydrophobic R-Group Elicit Abnormal Oligomerization of CoV N-CTD by Targeting CoV N-NTD

**FIGURE 3:**
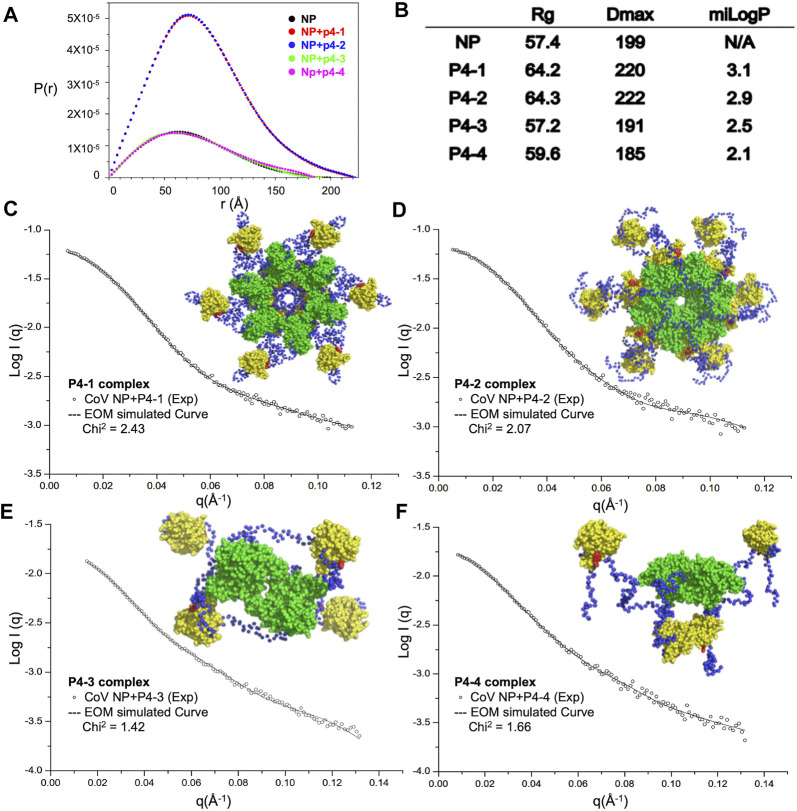
Increased hydrophobicity of P4 ligand is correlated with the aggregation tendency of CoV N protein. **(A)** SAXS analysis of full-length CoV N protein complexed with P4 derivative compounds. Normalized results from GNOM are described with pairwise distance distribution P(r) and maximum distance. **(B)** The calculated values of Rg, Dmax and miLogP of each complex. **(C–F)** (left) Scattering profiles of P4-1 complex **(C)**, P4-2 complex **(D)**, P4-3 complex **(E)**, and P4-4 complex **(F)** and normalization fitting with GNOM (dashed lines). (Right) Representative models of P4-1 complex **(C)**, P4-2 complex **(D)**, P4-3 complex **(E)**, and P4-4 complex **(F)** generated by CRYSOL simulations of SAXS data. NTD, CTD, linker and P4s are shown as yellow, green, blue and red, respectively.

To understand whether the designed modulators of N-NTD can elicit the abnormal aggregation of N protein, we purified full-length MERS-CoV N protein and the quality of protein samples were pure in solution (Supplementary Figure S1A). We examined the solution conformation of full-length MERS-CoV N protein in the presence of each compound by SAXS (Figure 3). Figure 3A shows the fitted distance distribution function of the N protein with or without each compound. The addition of P4-1 and P4-2 resulted in an increase in the radius of gyration (Rg) and maximum dimensions (Dmax) from 57 to 64 Å for P4-1 and from 199 Å to 220 Å for P4-2, respectively. In contrast, the addition of P4-3 and P4-4 did not change the value of Rg but caused a slight decrease in Dmax from 199 to 191 (P4-3) and 199 to 185 Å (P4-4). The results revealed that the size of MERS-CoV N protein in solution increased upon binding to ligands with more hydrophobic R-groups. Next, we employed an ensemble optimization method (EOM) with SAXS data and structures of the N-terminal domain (solved in this study) and C-terminal domain (CTD, PDB ID: 6G13) (Nguyen et al., 2019) to obtain the structural models for MERS-CoV N protein in complex with each ligand. The chosen ensemble fitted the data very well, and the representative structural models are shown in Figures 3C–F. In the presence of P4-1 and P4-2, the N proteins formed a dodecameric high-ordered structure, in which the CTD dimers aligned inward as a center ring. In each N protein dimer, the basic building block for CoV N protein, one NTD associated with the center ring formed by CTD, and the other NTD hung out of the ring in a monomer form (Figures 3C,D). However, the fitting conformation of the N protein with P4-3 or P4-4 was a tetramer, in which the two CTD dimers were placed at the center and the four NTD monomers were placed outside this center (Figures 3E,F). The conformation was similar to the previously solved structure of MERS-CoV N protein, indicating that P4-3 and P4-4 compounds are unable to induce the formation of higher-order oligomerization of full-length N protein (Supplementary Figure S2) (Lin et al., 2020).

### 2.4 Crystal Structure of the MERS-CoV N-NTD Complexed With P4 Compounds

We applied X-ray crystallography to elucidate the detailed mechanism employed by P4 compounds, while providing insights into the structural features required for a P4 compound design. We purified MERS-CoV N-NTD and further confirmed these sample were homogenous in solution by size-exclusive chromatography (Supplementary Figure S1B). We determined the structures of MERS-CoV N-NTD in complex with each ligand by molecular replacement (MR) using the crystal structure of HCoV-OC43 N-NTD as the search model (Chen et al., 2013). The complex structures of N-NTD with P4 compounds were solved at a resolution of around 2.5 Å (Supplementary Table S1). As shown in Supplementary Figure S2A, the N-NTDs of all the complexes shared a similar structural core containing a five-stranded antiparallel β-sheet sandwiched between loops. The conformation was organized into a right-handed, fist-shaped structure conserved across various CoVs (Nations, 2020) (Supplementary Figure S3B). A Additionally, the ligands were present alongside the protein (Supplementary Figure S3A). We then analyzed the detailed interaction between P4 compounds and N-NTD with LigPlot + suite (Laskowski and Swindells, 2011).

**FIGURE 4:**
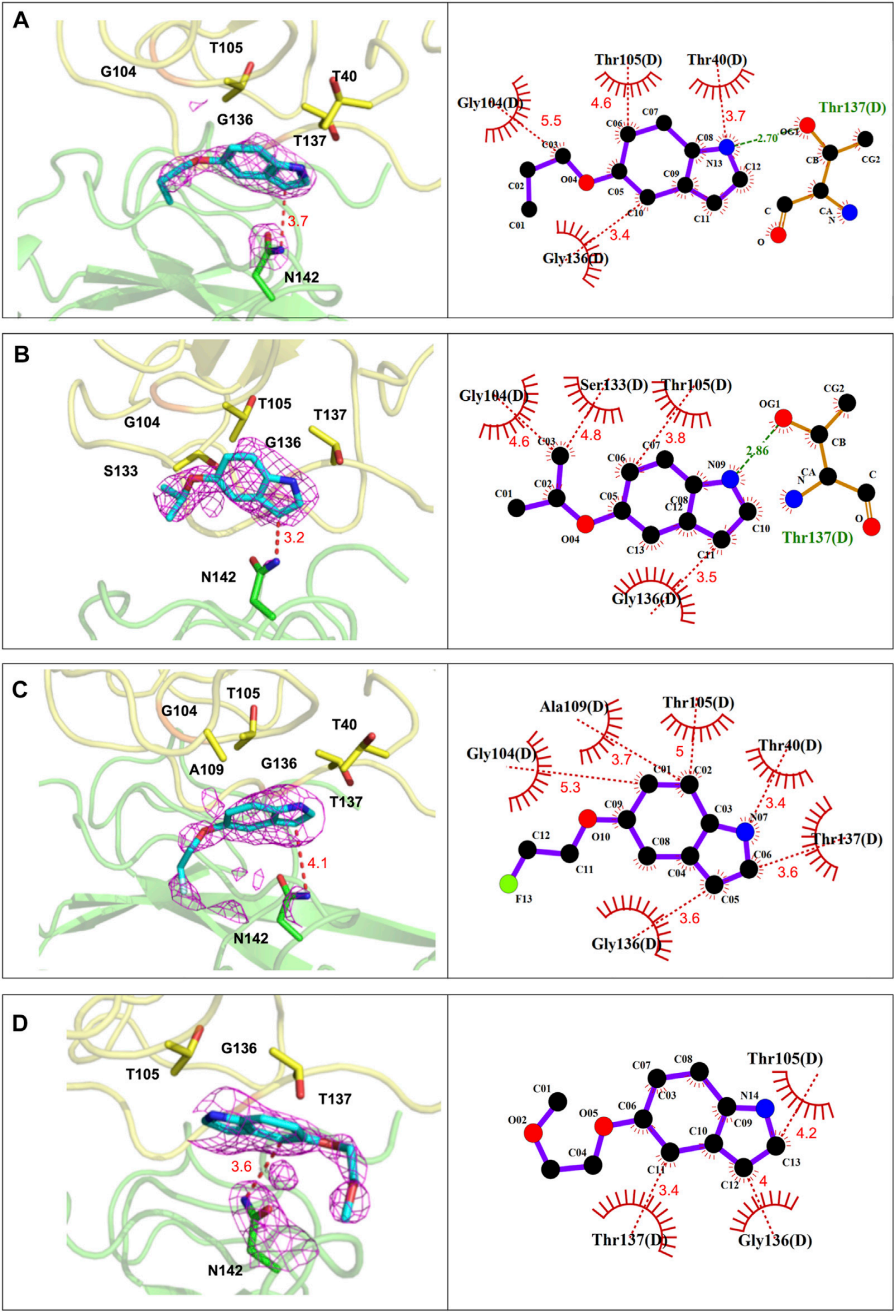
The detailed interactions between CoV N-NTD and each compound at P4-binding site. (Left) The CoV N protein was shown in cartoon and the residues involved in ligand binding were labeled and showed as sticks. (Right) Ligplot diagram of the interactions between N-NTD and P4 compounds. The contacting residues were labelled. Hydrophobic contacts and hydrogen bonds were displayed as red and green dashed lines, respectively. **(A)** The interactions of P4-1 complex. **(B)** The interactions of P4-2 complex. **(C)** The interactions of P4-3 complex. **(D)** The interactions of P4-4 complex.

As shown in Figure 4, the indole ring and the side chain of P4-1 contributed to hydrophobic contacts with T40, T105, G136, T137, and G104 of N-NTD, respectively. In addition, one hydrogen bond was formed between the pyrrole ring of the indoline moiety and the T137 of N-NTD. The P4-2 complex shares a nearly identical interaction composition with P4-1, in which the indole ring and the side-chain contributed hydrophobic contacts by packing against T105, G136, G104, and S133 of N-NTD, respectively. The hydrogen bond between the pyrrole ring of the indoline moiety and the T137 of N-NTD was also detected in the P4-2 complex. Relative to P4-1 and P4-2, the interactions between P4-3 and P4-4 with N-NTD were mediated by only the hydrophobic contacts, contributed by T40, G104, T105, A109, G136, and T137 and T105, G136, and T137 of N-NTD for P4-3 and P4-4, respectively.

These structural analyses revealed that the side chains of P4 compounds did not strongly participate in protein interactions. Therefore, to understand why these four compounds have different capacities to induce the aggregation of whole N protein, we further examined their drug-binding position. We found that the P4-contacting site is a related hydrophobic and flat surface that accommodates P4 compounds and influences the position of the indole moiety based on the hydrophobicity of the R groups (Supplementary Figure S4). In accordance with this notion, the positions of the indole moiety of P4-1 and P4-2 almost overlapped, while the position of the indole moiety of P4-3 was far from that of P4-1 and P4-2. This resulted in the distances between the contacting residues of N-NTD and P4-1 (∼3.98 Å) and P4-2 (∼3.91 Å) being moderately shorter than that of P4-3 (∼4.1 Å). This also affected the distance between the pyrrole ring of each compound and the side chain of T137 of N-NTD, leading to an important hydrogen bond formed between N-NTD and P4-1, P4-2, but not P4-3 (Supplementary Figure S4). The strength of the hydrophobic contacts was largely determined by the interacting distance. In addition, the strength of hydrogen bonds is generally stronger than that of hydrophobic contacts (Ferreira de Freitas and Schapira, 2017), which implies that P4-1 and P4-2 bind to N-NTD more strongly than P4-3, which could explain why only P4-1 and P4-2 can elicit oligomerization. Accordingly, the side chain of P4-4, the most hydrophilic R group, was ejected by this hydrophobic environment and extended in the opposite direction to point to a hydrophilic cluster, which also largely changed the position of the P4-4 compound (Supplementary Figure S4A). Since our structural analysis showed that the P4-1 and P4-2 have more interactions with MERS-CoV N-NTD, we used the fluorescent quenching assay to confirm their binding affinities. The values of binding constant (Kd) for MERS-CoV-N-NTD to P4-1, P4-2 is found to be 23.4 ± 4.94 µM and 13 ± 4.24 *µ*M respectively. Accordingly, titration of P4-4 to the protein showed no significant quenching suggesting weaker affinity (Supplementary Figure S5). These results suggest that the indole compounds could target a hydrophobic surface on N-NTD, in which the hydrophobicity of the R-group could affect the contact of indole compounds with the N protein, thus affecting the aggregation tendency of full-length N protein.

### 2.5 Ligand-Protein Surface Mediates the Interaction Between N-NTD and N-CTD

To uncover the mechanism of the allosteric modulation of full-length N protein by P4 compounds, we compared the differences in the surface properties of N-NTD upon binding of P4 compounds. We found that the 5-hydroxyindole moiety of P4 compound occupied one cavity on N-NTD to create a novel surface that we named as “ligand-protein surface” (Supplementary Figure S4B). We further found that, compared to those of P4-1 and P4-2, the side chains of P4-3 and P4-4 protruded out of the ligand-protein surface (Supplementary Figure S6). Based on the side-chain property of each compound, the results suggested that the newly created surfaces in the P4-1 and P4-2 complexes may be flatter and more hydrophobic than those of P4-3 and P4-4 (Supplementary Figure S7). The results of SAXS experiments indicated that one N-NTD was associated with the central aggregated N-CTD during ligand-induced modulating events. We thus speculated that the formation of the “ligand-protein surface” helps increase the interaction area of each P4-N-NTD complex to bind to N-CTD. To test this hypothesis, we examined the surface hydrophobicity of the centrally aggregated N-CTD in a higher-ordered N protein oligomer. As expected, in the solution model of full-length N protein with P4-1 and P4-2, the ligand-protein surface on N-NTD was close to the hydrophobic surface of the N-CTD ring (Supplementary Figure S8). Based on these observations, we proposed that the binding of P4 affects the surface properties, including the flatness and hydrophobicity of N-NTD, which allows the association of N-NTD with N-CTD. This further influences the interaction behavior of N-CTD and eventually results in the higher ordered oligomerization of the N protein.

### 2.6 Application of P4 Compounds in Targeting the SARS-CoV-2 N-NTD

Since the N protein is highly conserved within various CoVs, we inferred that it could be possible to apply our strategy to other CoV inhibitions. Recently, SARS-CoV-2, a novel coronavirus closely related to SARS-CoV, has elicited a worldwide outbreak of pneumonia and poses a high risk to global public health (Hui et al., 2020; Zhu et al., 2020). Many structural studies have been performed to accelerate drug development against SARS-CoV-2 (Jin et al., 2020a; Jin et al., 2020b; Gao et al., 2020; Kang et al., 2020; Wang et al., 2020; Wu et al., 2020), including the crystal structure of SARS-CoV-2 N-NTD (Kang et al., 2020). To assess the possibility of P4 acting on SARS-CoV-2 N proteins and subsequently inhibiting viral replication, we compared the structure of the P4-1 complex with that of SARS-CoV-2 N-NTD within the P4 binding region. The amino acids of SARS-CoV-2, corresponding to the P4-1 interacting residues of MERS CoV N-NTD, have almost identical conformation compared to MERS CoV N-NTD (Figure 6A). The multiple sequence alignment of the N-NTD region of various β-CoVs also indicates that the residues involved in P4 binding are highly conserved (Figure 6B). This implies that P4 can induce higher-order oligomerization of the SARS-CoV-2 N protein, suggesting the possible development of indole derivatives in COVID-19 treatments.

## 3 Discussion

For the last two decades, there have been three successive outbreaks of new lethal CoVs such as SARS, MERS, and SARS-CoV-2. All these epidemics have significantly impacted human health and the economy. Interestingly, they are all characterized as the β subgroup of CoVs, and their RNA genomes are inherently inclined to have higher mutation rates. Therefore, the development of new anti-CoV agents is urgently needed to control the illnesses caused by these viruses.

The N protein is the most abundant structural protein of CoVs, which is responsible for the formation of the RNP complex to facilitate viral replication and translation (Almazan et al., 2004; Zuniga et al., 2010). The N protein is highly conserved within various CoVs and has a lower mutation rate. These properties make N protein a promising target for the development of an antiviral agents. Two strategies have been used to inhibit the N protein function (Cianci et al., 2012). One was to develop antiviral compounds targeting the RNA binding site of the N protein (Lin et al., 2014; Tarus et al., 2015) and the other was to block the normal N protein oligomerization (Gerritz et al., 2011) to prevent the formation of the RNP complex during viral replication. In our recent study, we identified manipulation of a non-native PPI of MERS-CoV N-NTD by a lead compound (P3), as being a novel strategy for designing antiviral drugs (WHO, 2020). These results established a model for a PPI stabilizer targeting N-NTD for antiviral drug development.

In this study, we designed P4 compounds to elicit high-order oligomerization of N protein via a mechanism different from that of P3. Among all types of PPI modulators, the allosteric modulator is defined as a group of ligands that bind to the sites on a protein spatially far from the functional sites and induce a conformational change that influences the binding of the protein to its interacting partner(s) (Petta et al., 2016; Duan et al., 2019). Our crystal structures of P4 complexes revealed that P4 acts on a surface remote from the non-native PPI of the N-NTD dimer. The thermal-stability experiments also implied that P4 did not stabilize this non-native dimer (Supplementary Figure S9). Furthermore, the solution conformations of full-length N protein in the presence or absence of P4 compounds showed a significant difference, in which P4 largely altered the interacting behavior of N-CTD, resulting in the abnormal oligomerization of the N protein. Moreover, according to the solved structures of allosteric modulators in complex with their target proteins, the allosteric modulators always contain a hydrophobic scaffold accompanied by certain polar interactions (Duan et al., 2019). For instance, the crystal structure of CD40L in complex with its allosteric inhibitor, BIO8898, revealed that the binding pocket of BIO8898 is 80% hydrophobic and 5% polar (Silvian et al., 2011). In addition, the complex structure of hCdc34 and its allosteric stabilizer, CC0651, revealed that the contact between CC0651 and hCdc34 was dominated by hydrophobic interactions, and only one hydrogen bond was formed in this complex (Ceccarelli et al., 2011). These examples imply that the interaction of allosteric modulators may require the majority of hydrophobic contacts with the help of certain critical hydrogen bonds to strengthen the ligand-protein interaction, thereby affecting the overall conformations of the target proteins. Our structural analyses revealed that the interactions between N-NTD and P4 compounds were mainly mediated by hydrophobic contacts. Furthermore, the formation of one critical hydrogen bond plays an important role in this regulation. However, the detailed mechanism of P4-induced aggregation of N proteins remains unproven. Based on the above-mentioned definitions and properties of P4 compounds, we inferred that P4 functions through allosteric modulation.

Allosteric ligands possess a unique mechanism; they bind to the region far from the orthosteric site and thus do not need to compete with their PPI partners. This can reduce the dosage of allosteric modulators and offers them a promising feature in PPI-based drug development. However, due to the limited knowledge of allosteric regulation in PPI manipulation, discovering allosteric PPI modulators is still highly challenging. Similar to P4 compounds, most allosteric PPI modulators were discovered serendipitously. One famous example of allosteric PPI modulators against viruses is the allosteric IN inhibitor (ALLINI) (Wilson et al., 2019), a major class of novel inhibitors of HIV-1 integrase (IN), initially designed to inhibit the interaction of IN and its cellular substrate, but was later proven to promote IN multimerization, thus inhibiting IN activity. This suggests that allosteric modulators of protein oligomerization can be a strategically used to develop antiviral agents, and the results from our study could serve as the basis for allosteric PPI modulator design against CoV N protein.

In the past decade, fragment-based drug discovery (FBDD) has become a powerful tool for the discovery of lead compounds against pathogens. X-ray crystallography also plays a profound role in both drug discovery and fragment advancement. Recently, some drugs have been discovered using the FBDD approach combined with X-ray crystallography; these drugs are currently under safety and effectiveness evaluation in clinical trials (Wyatt et al., 2008; Howard et al., 2009). Hence, FBDD may be used as a powerful tool to facilitate drug development. To consolidate this approach, more detailed correlations between ligands and targets via structural techniques need to be included to enhance their progress. We also investigated the chemical properties required for the therapeutic effects of P4 compounds targeting the CoV N-NTD. Two chemical properties are crucial for the development of allosteric PPI modulators targeting CoV N-NTD. First, the indoline moiety, which not only serves as a hydrophobic core for acting on the targeting surface, but also provides an N-H site for hydrogen bond formation between the ligand and N-NTD. Second, a hydrophobic branching moiety is required to fine-tune the final position of the indoline core to initiate an interaction between the ligand and N-NTD. This information can be further utilized for new drug development via the FBDD approach.

**FIGURE 5:**
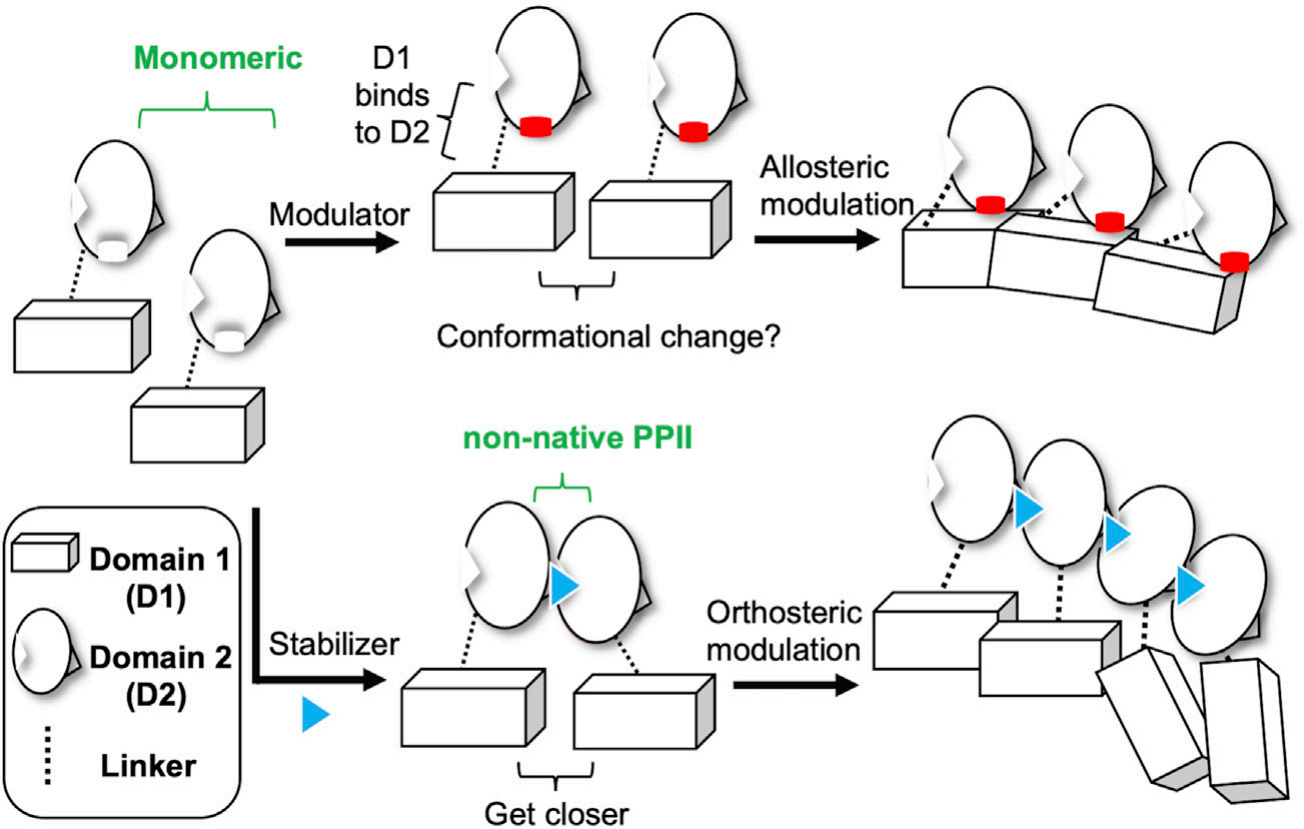
Two different mechanisms of small-molecules targeting CoV N-NTD for inducing abnormal oligomerization. Allosteric modulator binds to the CoV N-NTD to create a hydrophobic flat surface that effect the oligomeric tendency of N-CTD, which induces the abnormal oligomerization of N protein. Whereas, N-NTD stabilizer acts on the non-native interface of CoV N-NTD dimer, which reduces the distance between targeted N proteins and eventually results in the aggregation of whole N protein.

**FIGURE 6:**
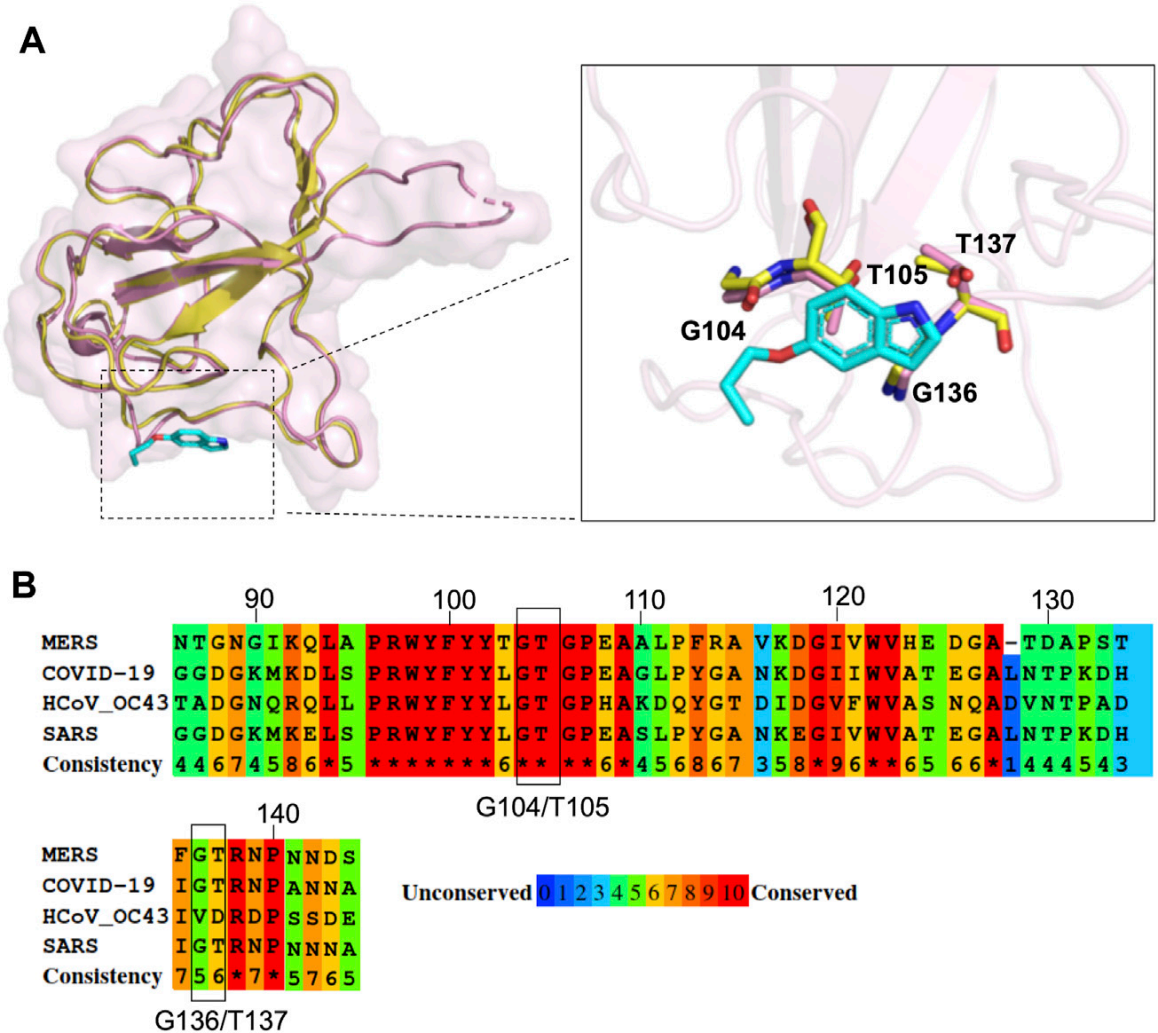
Structural comparison of MERS CoV N-NTD with that of SARS-CoV-2 at P4 binding surface. **(A)** The structure of P4-1:MERS CoV N-NTD complex (yellow) was aligned to SARS-CoV-2 N-NTD (PDB: 6M3M), the interacting residues are highlighted in right box. **(B)** Protein sequence alignment of N-NTD of various CoVs. The conservation scoring was calculated by PRALINE and indicated as 0 (least conserved) to 10 (most conserved). The residues involved in P4-1 binding were indicated by black box.

Finally, based on our recent studies and current results, we propose two different strategies of small molecules targeting CoV N-NTD to induce abnormal aggregation (Figure 5). One is to target the non-native interface of the N-NTD dimer, which functions as a stabilizer to drive the N protein to get closer, thus resulting in oligomerization of the N protein. The other is to act on one N-NTD, which serves as an allosteric modulator that affects the oligomerization behavior of N-CTD. Our results show that compounds P4-1 or P4-2 can induce the abnormal aggregation of the CoV N protein (Figure 3). We also found that P4-1 or P4-2 may act on other CoV N proteins, such as SARS-CoV-2, in addition to MERS-CoV, based on the results of structural comparison and sequence alignment (Figure 6).

Taken together, our current results provide the structural features of small molecules capable of eliciting high-order oligomerization of CoV N protein through an allosteric mechanism. Although it is not possible at this stage to demonstrate the ability of P4-1 and P4-2 to inhibit the potential propagation of coronaviruses, our study highlights an important strategy for manipulating the PPI’s by targeting viral proteins. Finally, these findings will accelerate the development of novel anti-CoV agents based on the manipulation of PPIs of the CoV N proteins.

## 4 Materials and Methods

### 4.1 Cloning, Protein Expression, and Purification

MERS-CoV N-NTD expression and purification were performed according to previously described methods (Wang et al., 2015). The cDNA fragments of MERS-CoV N39-165 protein were cloned into a pET-28a expression vector (Merck, Darmstadt, Germany) containing a histidine tag-encoding sequence. The pET-28a/MERS-CoV N-NTD construct was transformed into *Escherichia coli* BL21 (DE3) pLysS cells and the cells were grown in Luria Bertani (LB) broth containing 50 μg/ml kanamycin at 37°C until the optical density (OD) reached 0.6–0.8 at 600 nm. However, the full-length MERS CoV N protein was cloned into a pET-21a expression vector (Merck, Darmstadt, Germany) containing a histidine tag-encoding sequence. The transformed cells were grown in LB broth containing 50 μg/ml ampicillin for 5 h at 200 rpm and 37°C. Protein expression was induced by adding 1 mM isopropyl-β-D-thiogalactopyranoside (IPTG), followed by incubation at 10°C at 200 rpm for 24 h. The bacteria were harvested via centrifugation (8,000 rpm, 12 min, 4°C), and the bacterial pellets were resuspended in the lysis buffer (50 mM Tris–HCl pH 7.5, 150 mM sodium chloride (NaCl), 15 mM imidazole, 1 mM phenylmethylsulfonyl fluoride (PMSF)) and sonicated on ice. The soluble protein was then obtained from the supernatant after centrifugation (13,000 rpm, 40 min, 4°C). The MERS-CoV N-NTD protein was purified using a nickel-nitrilotriacetic acid (Ni-NTA) column (Merck, Darmstadt, Germany) with an elution gradient ranging from 15 to 250 mM of imidazole. Pure fractions were collected and dialyzed against a low-salt buffer and concentrated using a filter device with a 3 kDa cutoff membrane (Merck, Darmstadt, Germany). Protein concentrations were determined using the Bradford method (Wang et al., 2015).

### 4.2 Compound Design

To start the fragment search, the crystal structure of MERS-CoV N-NTD was used as a template to target the B-site of the non-native dimerization interface with a PPI fragment library and the LibDock software. According to the filtering criterion described in the main text, the chosen small molecule would serve as a starting fragment and further be applied to similarity searches using the ZINC database. The derivatives of the selected compound with the desired R-group modification were further synthesized as described below.

### 4.3 Synthesis of P4 Compounds

#### 4.3.1 5-(Propoxy)-1H-Indole (P4-1)

Sodium hydride (66 mg, 1.65 mmol) and 5-hydroxyindole (200 mg, 1.50 mmol) were dissolved in 2.4 ml of dimethylformamide (DMF) and stirred at 25°C for 30 min, followed by the addition of 1-iodopropane (0.16 ml, 1.65 mmol) and stirred at 25°C for 3 h. The reaction mixture was then extracted with EtOAc (15 × 3 ml) and combined organic layers, which were washed with brine, dried over magnesium sulfate (MgSO_4_), and concentrated. The residue was purified by column chromatography on silica gel to obtain compound **P4-1** (199 mg, 76%) as a brown oil. R_*f*_ 0.29 (EtOAc/Hex = 1/10); [α]^25^_D_ +30.33 (*c* 0.1, DCM); IR (NaCl) *v* 3,443, 3,039, 2,860, 1,625 cm^−1^; 1H NMR (400 MHz, CDCl3) δ 8.05 (s, 1H), 7.27 (t, J = 8.0 Hz, 1H), 7.18 (s, 1H), 7.11 (d, J = 2.4 Hz, 1H), 6.89 (d, J = 2.4 Hz, 1H), 6.47 (m, 1H), 3.97 (t, J = 8.0 Hz, 2H), 1.83 (m, 2H), 1.06 (t, J = 8.0 Hz, 3H); 13C NMR (400 MHz, CDCl3) δ 153.2, 130.9, 128.1, 124.9, 112.6, 111.7, 103.4, 101.8, 70.4, 22.6, 10.4; HRMS (ESI, M + Na+). The calculated MW for C11H13NONa was 198.0895, whereas the actual value was 198.0897. The 1H and 13C nuclear magnetic resonance (NMR) spectral data of compound P4-1 were consistent with previously published literature (Unzue et al., 2016).

First, 5-hydroxyindole (500 mg, 3.76 mmol) was dissolved in 5 ml of DMF along with 2-bromopropane (0.42 ml, 4.51 mmol) and sodium hydride (300 mg, 7.51 mmol), and stirred at 60°C. After 6 h, 2-bromopropane (0.71 ml, 7.51 mmol) and sodium hydride (300 mg, 7.51 mmol) were added and it was stirred at 60°C for 3 h. The reaction mixture was extracted with EtOAc (20 × 3 ml), and the combined organic layers were washed with brine and dried over MgSO_4_. The residue was purified by column chromatography on silica gel to give the desired compound **P4-2** (5-Isopropoxy-1H-indole) (334 mg, 51%) as a yellow oil. R_*f*_ 0.44 (EtOAc/Hex = 1/4). [α]^25^_D_ +57.59 (*c* 0.1, DCM); IR (NaCl) *v* 3,443, 3,039, 2,860, 1,625 cm^−1^; ^1^H NMR (400 MHz, CDCl3) δ 8.05 (s, 1H), 7.28 (d, J = 8.8 Hz, 1H), 7.18 (t, J = 2.8 Hz, 1H), 7.14 (d, J = 2.4 Hz, 1H), 6.86 (dd, J = 4.4, 1.2 Hz, 1H), 6.47 (m, 1H), 4.52 (m, 1H), 1.36 (d, J = 4.0, 6H), 1.35 (s, 3H); ^13^C NMR (400 MHz, CDCl3) δ 152.0, 131.2, 128.3, 124.9, 114.4, 111.6, 106.4, 102.2, 71.4, 22.2; HRMS (ESI, M + Na^+^). The calculated MW for C11H13NONa was 198.0895, whereas the actual MW was 198.0898. The 1H and 13C NMR spectral data of compound P4-2 were consistent with previously published literature (Taydakov et al., 2011).

#### 4.3.2 5-(2-Fluoroethoxy)-1H-indole (P4-3)

First, 2-bromoethanol (1.14 ml, 16.00 mmol) was added dropwise to diethylaminosulfur trifluoride (2.10 ml, 15.90 mmol) in diglyme (10 ml) at –50°C. The reaction mixture was heated to 25°C and stirred for 1 h. The most volatile portion was distilled at 70°C, and the residue was used in the next step of the reaction without further purification. Then, 5-hydroxyindole (200 mg, 1.50 mmol) in acetone (2 ml) was added to the solution of 1-bromo-2-fluoroethane (286 mg, 2.25 mmol) and K_2_CO_3_ (415 mg, 3.00 mmol) in DMF. The reaction mixture was heated under reflux and stirred for 6 h and then extracted with EtOAc (15 × 3 ml). The combined organic layers were washed with brine and dried over MgSO_4_. The residue was purified by column chromatography on silica gel to give the desired compound **P4-3** (66 mg, 24% in 2 steps) as a yellow oil. R_*f*_ 0.35 (EtOAc/Hex = 1/4). [α]^25^_D_ +22.27 (*c* 0.1, DCM); IR (NaCl) *v* 3,443, 3,039, 2,860, 1,625 cm^−1^; ^1^H NMR (400 MHz, CDCl3) δ 8.08 (s, 1H), 7.30 (d, J = 8.8 Hz, 1H), 7.20 (t, J = 2.8 Hz, 1H), 7.13 (d, J = 2.4 Hz, 1H), 6.91 (dd, J = 4.4, 1.2 Hz, 1H), 6.49 (m, 1H), 4.84 (t, J = 4.4 Hz, 1H), 4.72 (t, J = 4.4 Hz, 1H), 4.30 (t, J = 4.0 Hz, 1H), 4.23 (t, J = 4.0 Hz, 1H); ^13^C NMR (400 MHz, CDCl3) δ 124.9, 113.0, 111.8, 103.9, 102.4, 83.1, 81.4, 68.2.; HRMS (ESI, M + Na^+^). The calculated MW for C10H10FNONa was 202.0644, whereas the actual MW was 202.0690. The 1H and 13C NMR spectral data for compound **P4-3** were consistent with previously published literature (Wang et al., 2017).

#### 4.3.3 5-(2-Methoxy-ethoxy)-1H-indole (P4-4)

To a solution of sodium iodide (2.71 g, 18.1 mmol) in acetone (15.6 ml), 2-chloroethyl methyl ether (0.78 ml, 8.60 mmol) was added. The mixture was then heated in reflux and stirred. After 16 h, the mixture was filtered and concentrated. The residue was used in the next reaction step, without further purification. Sodium hydride (66 mg, 1.65 mmol) was dissolved in 1.2 ml of DMF, and the mixture was slowly added with 5-hydroxyindole (200 mg, 1.50 mmol) in DMF (1.2 ml) and stirred at 25°C. After 30 min, 2-methoxyethyl iodide (363 mg) was added to the mixture and stirred continuously at 25°C for 3 h. The resulting mixture was washed with brine and the aqueous phase was extracted with EtOAc (5 × 50 ml). The combined organic layers were dried over MgSO_4_, filtered, and concentrated under reduced pressure. The residue was purified by column chromatography on silica gel to give the desired compound **P4-4** (117 mg, 41% in 2 steps) as a brown oil. R_*f*_ 0.60 (EtOAc/Hex = 1/1). [α]^25^_D_ +21.30 (*c* 0.1, DCM); IR (NaCl) *v* 3,443, 3,039, 2,860, 1,625 cm^−1^; 1H NMR (400 MHz, CDCl3) δ 8.07 (s, 1H), 7.28 (d, J = 8.0 Hz, 1H), 7.19 (d, J = 2.4 Hz, 1H), 7.13 (d, J = 2.4 Hz, 1H), 6.93 (m, 1H), 6.48 (m, 1H), 4.17 (m, 2H), 3.77 (m, 2H), 3.29 (s, 3H); ^13^C NMR (400 MHz, CDCl3) δ 153.1, 131.1, 128.1, 124.9, 112.9, 111.7, 103.6, 102.2, 71.2, 68.0, 59.0; HRMS (ESI, M + Na^+^). The calculated MW for C11H13NO2Na was 214.0844, whereas the actual MW was 214.0841.

### 4.4 Crystallization and Data Collection

Each MERS-CoV N-NTD:P4 complex was crystallized using the sitting-drop vapor diffusion method. The crystals of MERS-CoV N-NTD:P4 complex were obtained from a solution of 5 mg/ml MERS-CoV N-NTD, 25 mM Tris-HCl (pH 7.5), 75 mM NaCl, 140 mM 2-(N-morpholino) ethanesulfonic acid (MES) (pH5.5), 75 mM ammonium sulfate ((NH_4_)_2_SO_4_), 29% polyethylene glycol (PEG) 3,350, 2 mM sodium bromide (NaBr), and 2 mM P4 ligand equilibrated against 300 μL of reservoir solution consisting of 280 mM MES (pH 5.5), 150 mM (NH_4_)2SO4, 58% PEG 3350, and 4 mM NaBr at 20°C. The crystals were harvested after 3 weeks. Diffraction datasets for the MERS-CoV N-NTD in complex with P4-1∼P4-3 were collected at beamline 05A1 of the Taiwan Photon Source (TPS) of the National Synchrotron Research Center (NSRRC; Hsinchu City, Taiwan). Diffraction of the MERS-CoV N-NTD:P4-4 complex was performed at the beamline 15A1 of Taiwan Light Source (TLS). All the crystals were mounted directly on the loops without use of cryoprotectant.

### 4.5 Structural Determination and Refinement

All diffraction data were processed and scaled using the HKL2000 software. The phases were solved by molecular replacement (MR) in PHENIX (version 1.10.1) (Adams et al., 2002) using HCoV-OC43 N-NTD (PDB ID: 4J3K) as the search model. According to the electron density map, the structures were adjusted and refined using WinCoot (version 0.8.4) (Emsley et al., 2010) and PHENIX until the R-factor and R-free values decreased. The crystallographic and refinement statistics of the MERS-CoV N-NTD/P4 ligands complexes are listed in Table 1. The refined structures were visualized using PyMOL (version 1.8) (Kudlicki et al., 2007). The hydrophobic interactions between MERS-CoV N-NTD and P4 ligands were analyzed using LigPlot^+^ (Laskowski and Swindells, 2011). The distances of hydrophobic contacts between N-NTD and each compound were defined as the length between the nearest atom of the contacting residues and each compound, which were calculated using PyMOL.

### 4.6 Small-Angle X-Ray Scattering Experiments

SAXS data were collected using a monochromatic X-ray beam (*λ* = 0.828 Å) at the BL23A beamline coupled with a high-performance liquid chromatography (HPLC) system fitted with an Agilent-Bio SEC-3 300 Å column (Agilent Technologies, Santa Clara, United States) at the NSRRC (Hsinchu City, Taiwan). Protein samples (44 μM of MERS-CoV N) were incubated with ligands (440 μM) in a buffer containing 50 mM Tris-HCl (pH 8.5) and 150 mM NaCl for 1 h on ice. Then, a 300 μL aliquot was injected into the column at a flow rate of 0.02 ml/min and directed into a quartz capillary (2 mm diameter) For SAXS measurement, with X-rays and a sample-to-detector distance of 2.5 m, the scattering vector q, defined as q = (4π/λ) sinθ, with the scattering angle 2θ during a 30 s exposure, 36 frames of all samples were collected and merged, and analyzed to determine the initial *Rg* in PRIMUS (Version 3.1). The *D*_max_ and *P(r)* distance distributions were calculated from the experimental scattering curve using GNOM (version 4.1). EOM was used with the EMBL Hamburg web interface (Petoukhov et al., 2012). The crystal structures of the CTD domain of MERS-CoV N protein (PDB ID: 6G13) (Nguyen et al., 2019), MERS-CoV N-NTD (PDB ID: 4UD1) (Papageorgiou et al., 2016) and MERS-CoV N-NTD/P4 ligands (solved in this study) were used as rigid bodies in EOM analysis (Kelley et al., 2015). For EOM analysis, numerous models were generated at the beginning (structural pool). The EOM, fitting the experimental scattering curve with a linear combination, was selected from the structural pool. The presented conformations of P4 complexes were chosen because their ensemble-generated curves fit best to the experimental results of SAXS.

### 4.7 Protein Sequence Alignment and Structure Prediction

Protein sequence alignment of N-NTDs of various CoVs was performed using PRALINE. Sequences of all CoV N proteins have been deposited in Genbank with ACCESSION numbers of ASU90457.1, QHO62115.1, QBP84763.1 and AYV99827.1 for MERS-CoV, SARS-CoV-2, HCoV-OC43, and SARS-CoV, respectively. For the dimeric SARS-CoV-2 N-NTD, the SARS-CoV-2 N-NTD (PDB ID: 6M3M) structures were superimposed onto each monomer of the MERS-CoV N-NTD dimer, and the PDB file was exported using the PyMOL.

### 4.8 Accession Numbers

PDB ID: 6LNN (N:P4-1 complex), 7DYD (N:P4-2 complex), 6LZ6 (N:P4-3 complex) and 6LZ8 (N:P4-4 complex).

SASBD ID: SASDNF6 (N:P4-1 complex), SASDNG6 (N:P4-2 complex), SASDNH6 (N:P4-3 complex), SASDNI6 (N:P4-4 complex).

## Data Availability

The datasets presented in this study can be found in online repositories. The names of the repository/repositories and accession number(s) can be found in the article/Supplementary Material.

## Abbreviations:

CoV, Coronaviruses; MERS-CoV N-NTD, N-terminal domain of the MERS-CoV nucleocapsid protein; FBDD, Fragment-based drug design; SARS, Severe acute respiratory syndrome; MERS, Middle East respiratory syndrome; COVID-19, Coronavirus disease 2019; N, Nucleocapsid; E, Envelope; M, Matrix; S, Spike; IDRs, Intrinsically disordered regions; NTD, N-terminal RNA-binding domain; CTD, C-terminal dimerization domain; RNP, Ribonucleoprotein; PPI, Protein-protein interaction; ALLINI’s, Allosteric IN Inhibitors; IN, Integrase; DMF, Dimethylformamide; MR, Molecular replacement; SAXS, Small-angle X-ray scattering; Rg, Radii of gyration; Dmax, Maximum dimensions; EOM, Ensemble optimization method; IPTG, Isopropyl-β-D-thiogalactopyranoside; PMSF, Phenylmethylsulfonyl fluoride; Ni-NTA, Nickel-nitrilotriacetic acid; NMR, Nuclear magnetic resonance; MES, 2-(N-morpholino) ethanesulfonic acid; PEG, Polyethylene glycol; HPLC, High-performance liquid chromatography.
