# Corrigendum: Targeting the N-terminus domain of the coronavirus nucleocapsid protein induces abnormal oligomerization *via* allosteric modulation

**DOI:** 10.3389/fmolb.2022.1036858

**Published:** 2022-10-10

**Authors:** Jia-Ning Hsu, Jyun-Siao Chen, Shan-Meng Lin, Jhen-Yi Hong, Yi-Jheng Chen, U-Ser Jeng, Shun-Yuan Luo, Ming-Hon Hou

**Affiliations:** ^1^ Institute of Genomics and Bioinformatics and Department of Life Sciences, National Chung Hsing University, Taichung, Taiwan; ^2^ Department of Chemistry, National Chung Hsing University, Taichung, Taiwan; ^3^ National Synchrotron Radiation Research Center, Department of Chemical Engineering, National Tsing Hua University, Hsinchu, Taiwan

**Keywords:** PPI-based drug design, N protein, allosteric modulator, COVID-19, MERS-CoV

In the original article, there was an error in the Funding statement. We neglected to include the funder Ministry of Science and Technology (MOST). The correct Funding statement appears below:

“This work was supported by Ministry of Science and Technology (MOST) (MOST 109-2311-B-005-007-MY3, MOST 109-2628-M-005-001-MY4, MOST 109-2327-B-005-005) and ENgineering in Agriculture Biotech LEadership (ENABLE) (ENABLE Center 109ST001C).”

Additionally, there was an error in Accession Numbers. Instead of “6LZ5 (N:P4-2 complex)”, it should be “7DYD (N:P4-2 complex)”.

The corrected Accession numbers appears below:

“PDB ID: 6LNN (N:P4-1 complex), 7DYD (N:P4-2 complex), 6LZ6 (N:P4-3 complex) and 6LZ8 (N:P4-4 complex).

SASBD ID: SASDNF6 (N:P4-1 complex), SASDNG6 (N:P4-2 complex), SASDNH6 (N:P4-3 complex), SASDNI6 (N:P4-4 complex).”

The authors apologize for this error and state that this does not change the scientific conclusions of the article in any way. The original article has been updated.

