# Supplementary material for: Targeting the N-Terminus Domain of the Coronavirus Nucleocapsid Protein Induces Abnormal Oligomerization via Allosteric Modulation
Source: Front Mol Biosci. 2022 Apr 19;9:871499. doi: 10.3389/fmolb.2022.871499 (PMC9061996; doi:10.3389/fmolb.2022.871499)
Supplement: Supplementary file 1 [file DataSheet1.pdf]

Table S1. Crystallographic data collection and refinement statistics

Datasets	P4-1 complex	P4-2 complex	P4-3 complex	P4-4 complex
**Data collection**				
Beamline	NSRRC, TPS05A	NSRRC, TPS05A	NSRRC, TPS05A	TLS15A, NSRRC
Wavelength (Å)	1.0000	1.0000	1.0000	1.0000
Space group	$P2_1$	$P2_1$	$P2_1$	$P2_1$
**Unit cell dimensions**				
a, b, c (Å)	a=35.395<br>b=108.721<br>c=91.364	a=35.638<br>b=109.370<br>c=92.179	a=35.146<br>b=109.577<br>c=91.721	a=35.154<br>b=111.029<br>c=92.260
α, β, γ (°)	α=γ=90<br>β=101.057	α=γ=90<br>β=101.183	α=γ=90<br>β=100.914	α=γ=90<br>β=100.968
Resolution (Å)	30.00-2.64<br>(2.69-2.64)	30.00-2.38<br>(2.47-2.38)	30.00-2.65<br>(2.70-2.65)	30.00-2.45<br>(2.49-2.45)
Completeness (%)	98.9 (98.3)	98.8 (98.2)	98.7 (98.3)	96.7 (98.0)
Average I/σ	15.914 (4.062)	20.952 (4.5)	14.595 (5.314)	24.731 (5.175)
R_{merge} (%)	0.102 (0.376)	0.094 (0.458)	0.088 (0.301)	0.136 (0.358)
Redundancy	5.8 (5.6)	7.6 (7.1)	4.1 (4.0)	5.71 (2.3)
**Refinement**				
R_{work}/R_{free}	0.20/0.24	0.25/0.29	0.21/0.25	0.23/0.28
Number of atoms	3884	3909	3905	3869
Macromolecules	3762	3757	3771	3731
Ligands	13	13	13	14
Solvent	109	139	121	124
Average B-factor	52.65	40.09	48.69	42.25
Macromolecules	52.76	40.22	48.85	42.31
Ligands	65.01	54.14	69.42	64.18
**RMSD**				
Bond lengths	0.008	0.005	0.006	0.008
Angles (°)	1.19	1.03	1.23	1.25
**Ramachandran plot**				
Favored (%)	98.07	98.08	98.08	98.5
Outliers (%)	0	0	0	0
Clash score	10.07	13.5	10.32	13.18

Supplementary data

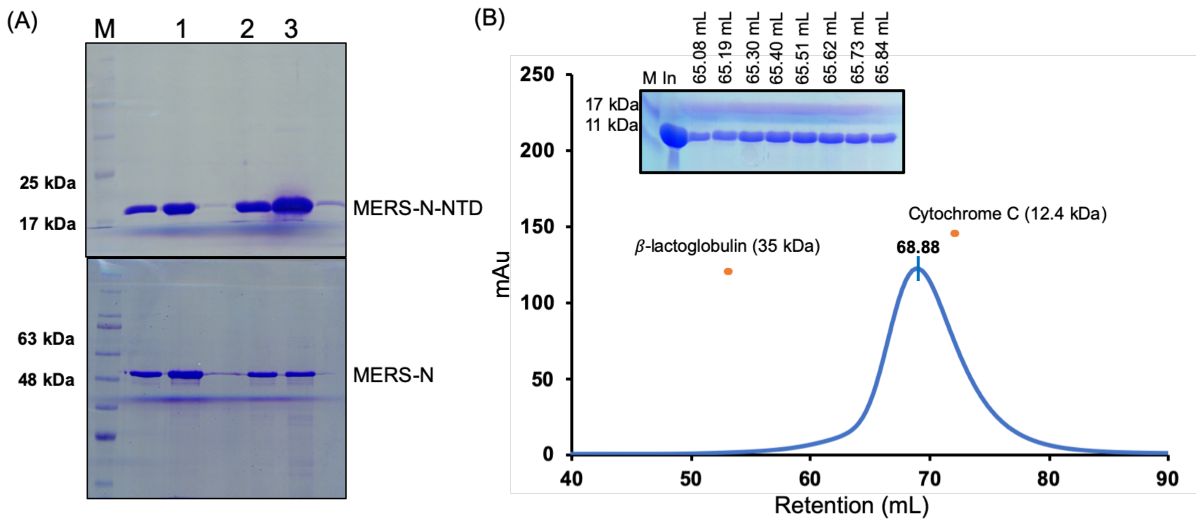

Figure S1. To express and purify the homogenous MERS-CoV N and MERS-CoV N-NTD protein in solution.

(A) The purification of full-length MERS-CoV N and MERS-CoV N-NTD protein in solution. Lane 1, 2, 3 represent as protein sample before dialysis, after dialysis and the concentrated sample. (B) The MERS-CoV N-NTD were subjected to the size-exclusive chromatography (SEC) by using Sephacryl S-100 High Resolution HiPrep 16/60 (GE Healthcare) and a single peak was eluted at the retention volume of 68.88 mL which was corresponding to around 15.71 kDa. According to the SEC profile of MERS-N-NTD, the results confirm the MERS-CoV N-NTD is pure and homogenous in solution. M: protein marker; In: the sample before injection.

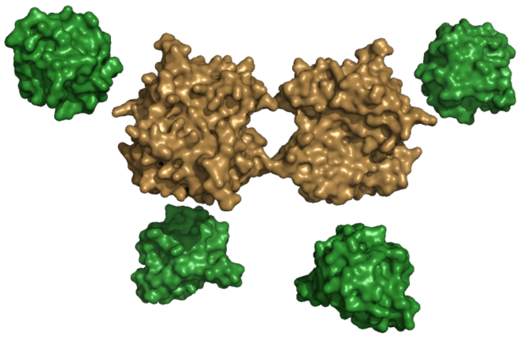

Figure S2. Representative models of the MERS-CoV N protein.

This model was generated by CRYSOL simulations of the SAXS data. The NTD and CTD are shown as green and brown, respectively (Lin et al., 2020).

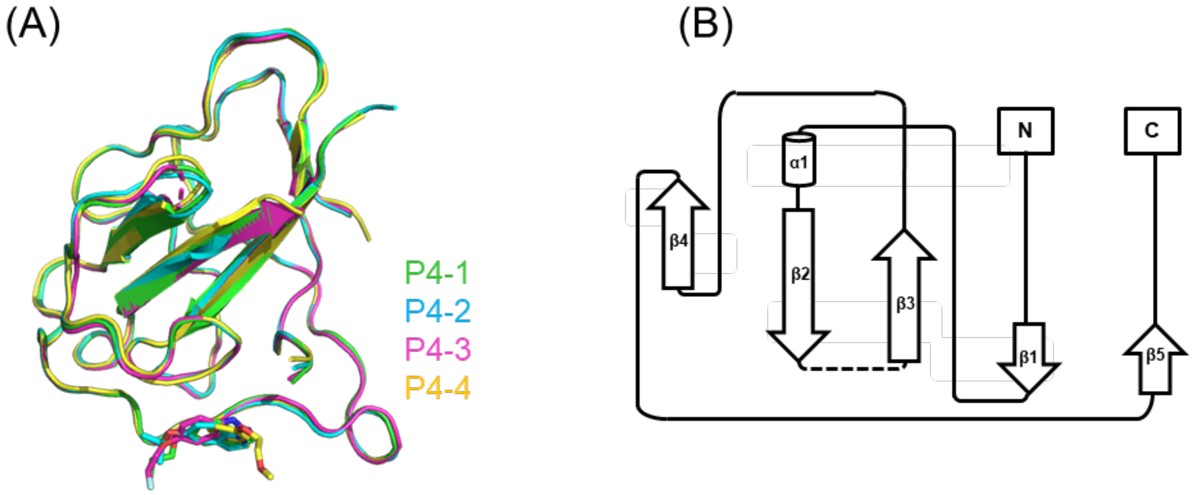

Figure S3. Overall structures of CoV N-NTD complexed with P4 compounds.

(A) Ribbon representation of CoV N-NTD complexes with each compound depicted as a stick structure. Superimposition of each co-complex shows high structural identity with RMSD at around 0.3 Å. (B) Topology diagram of the core structure of CoV N-NTD.

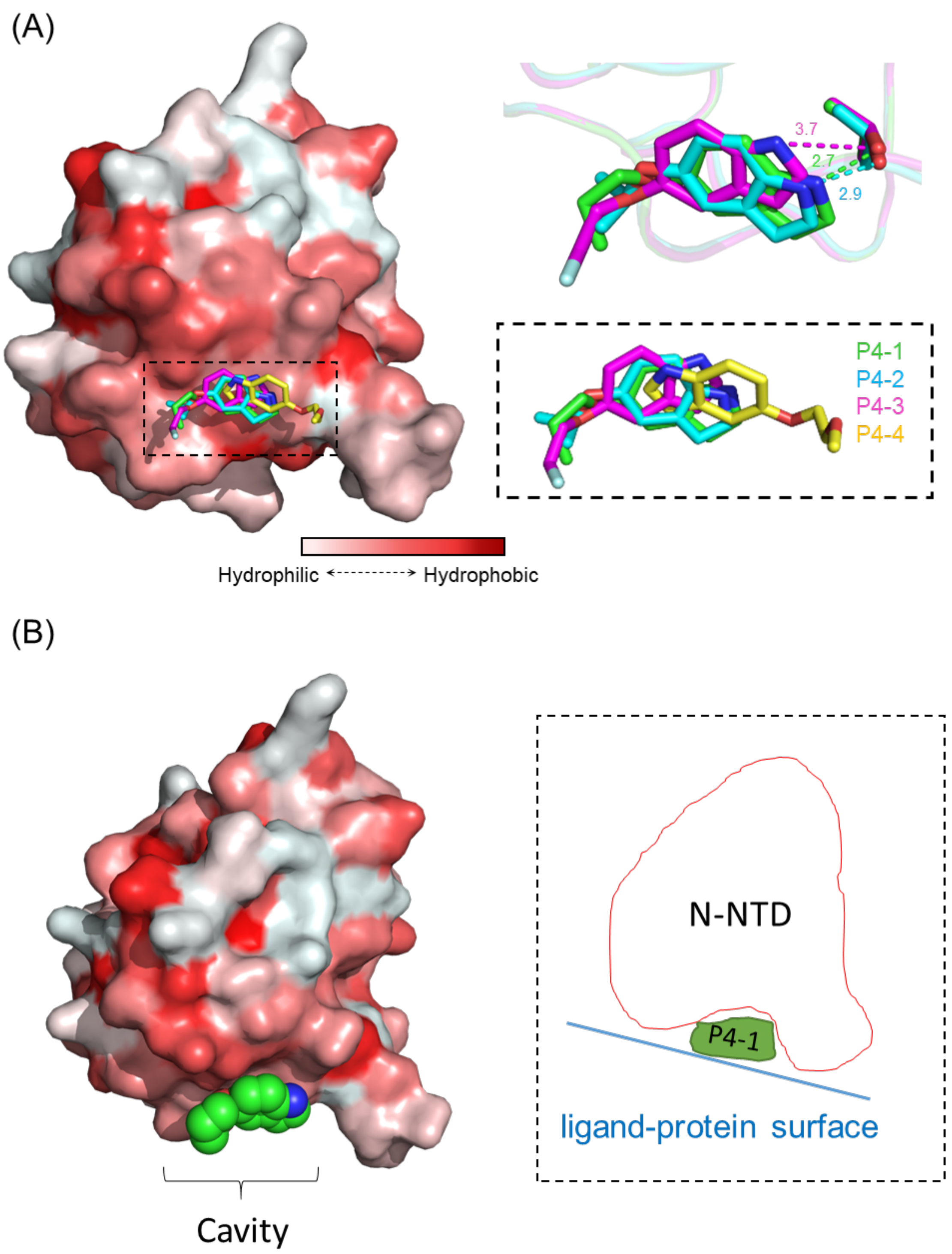

Figure S4. Structures of CoV N-NTD:P4 complexes.

N-NTD was shown in surface with the color according to hydrophobicity. The positions of each compound are highlighted in the black dotted box. **(B left)** Same as (A) except that only the P4-1 is shown with sphere to emphasize the novel surface formed by compound binding. **(B right)** Graph representation to show the formation of ligand-protein surface.

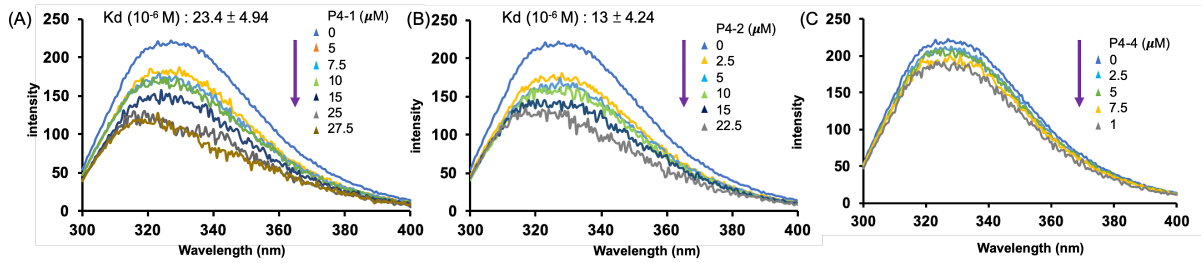

Figure S5. Fluorescence titration of P4-1, P4-2 and P4-4 to MERS-CoV-N-NTD .

MERS-CoV-N-NTD (1 μM) was dissolved in 20 mM Tris-HCl (pH 8.0) and 150 mM NaCl. The arrow indicated the spectra changes that induced by addition of compounds. Fluorescence quenching assays were performed by varies ligands (A) P4-1 (B) P4-2 and (C) P4-4. The MERS-CoV-n-nTD and P4 series compounds were excited at 280 nm and emission at 300-400 nm.

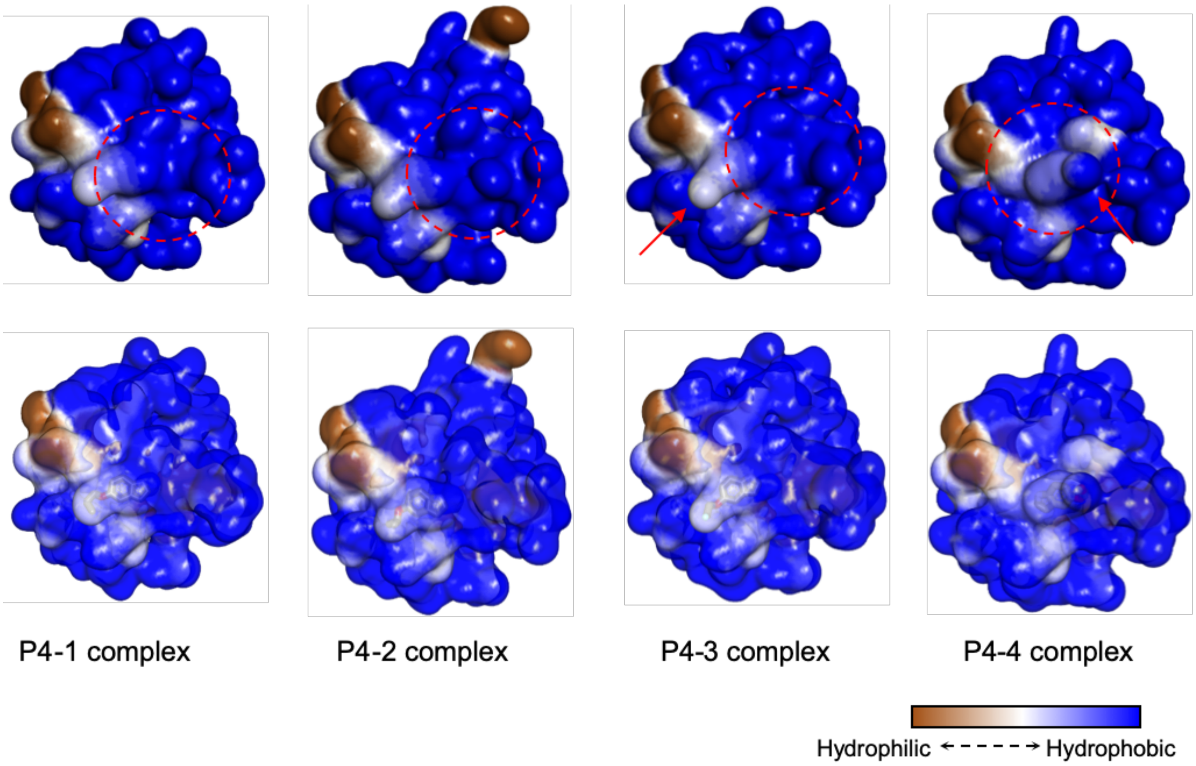

Figure S6. The hydrophobicity of CoV N-NTD:P4 complexes.

The whole complexes were shown with the surface representation according to hydrophobicity. The ligand-protein surfaces created by the binding of P4 derivatives were indicated with red dotted cycles. The protruded side chains of P4-3 and P4-4 were pointed out with red arrows.

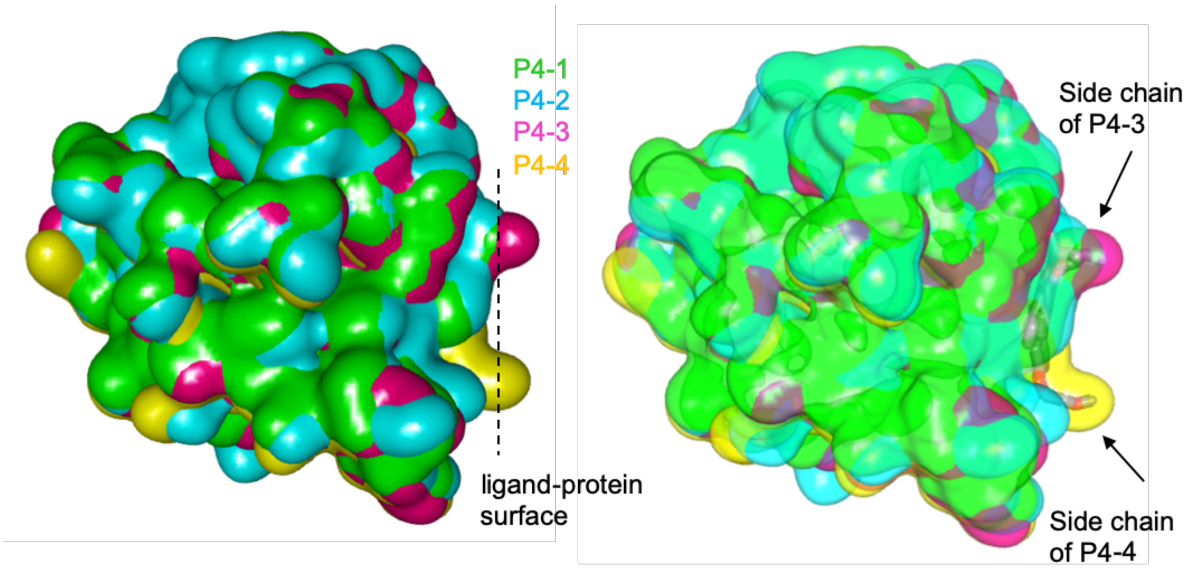

Figure S7. Ligand-protein surfaces created by the binding of P4 derivatives.

Superimposition of each co-complex with surface representation to show the side chains of P4-3 and P4-4 protrude out of the ligand-protein surfaces. The structures were colored with green, cyan, pink and yellow for P4-1 to P4-4, respectively.

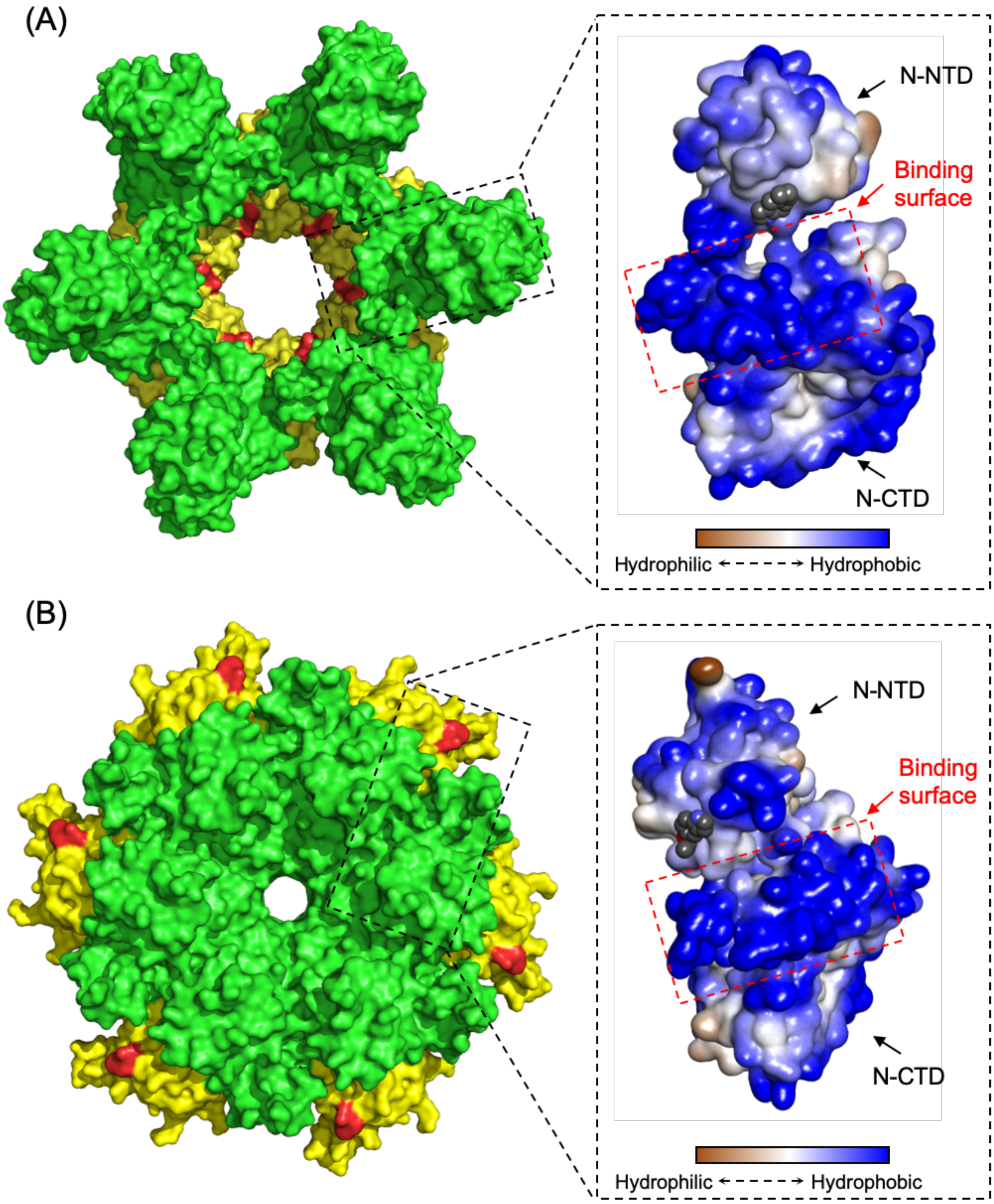

Figure S8. The central rings of the solution structures of full N protein in the present of P4-1 compound (A) or P4-2 compound (B).

(Left) The N-NTDs, N-CTDs and ligands were showed with yellow, green and red surface, respectively. The interacting interface of N-NTD and N-CTD was highlighted in right box. (Right) The protein part was shown in surface with the color according to hydrophobicity. The ligands were showed with sphere. The red box indicated the hydrophobic surface on N-CTD, expected for N-NTD binding.

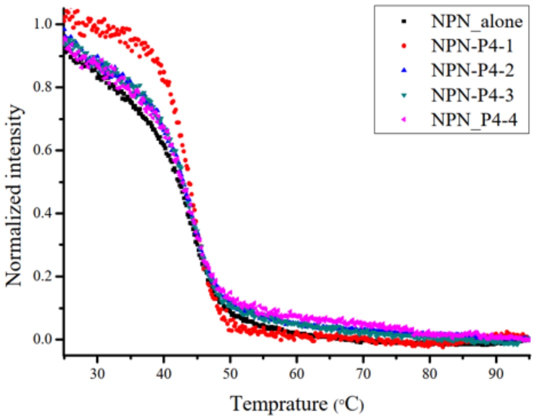

MERS-CoV N-NTD					
Ligands	DMSO	P4-1	P4-2	P4-3	P4-4
Tm (℃)<br>(ΔTm)	44.67±0.16<br>(0)	44.19±1.13<br>(-0.45±1.26)	44.45±1.11<br>(-0.32±1.23)	45<br>(0.5)	44.95±0.46<br>(0.3±0.45)

Figure S9. The effects of P4 compounds on the thermal-stability of CoV N-NTD.

The thermal-stability experiments were performed based on FL spectra of NTD (1 μM) incubated with P4-1−P4-4 (5 μM) for 1 h with a buffer containing 50 mM Tris-HCl (pH 7.5) and 150 mM NaCl. Data are shown as mean ± SD (n=3).

Materials and methods

Thermostability Measurements.

Thermostability assays were performed by using JASCO FP-8300 fluorescence spectrometer (JASCO International Co. Ltd., Tokyo, Japan) in 50 mM Tris-HCl (pH 7.5) and 150 mM NaCl. 1 μM N protein was incubated either with the control buffer or each compound (5 μM) at 4 °C for 2 h. UV absorbance vs temperature profiles were obtained by ramping the temperature from 4−95 °C at a 1 °C min−1 and recording the absorbance at 280 nm every 0.5 min.

Fluorescence quenching assay

Fluorescence spectra were recorded from 300 nm to 450 nm with tryptophan excitation at 280 nm. Titrations were performed by titrating 0.1 μL of each compounds into protein (1 μM) in 20 mM Tris-HCl (pH 8.0) and 150 mM NaCl until the saturation was reached. The binding constant (K_a) of the interaction between P4 series drug and MERS-N-NTD could be calculated according to a double logarithmic equation: log F0-F/F=logK+nlog[Q]. The dissociation constant (Kd) is 1/Ka. Average of two trials are reported.

Reference

Lin, S.-M., Lin, S.-C., Hsu, J.-N., Chang, C.-k., Chien, C.-M., Wang, Y.-S., et al. (2020). Structure-Based Stabilization of Non-native Protein–Protein Interactions of Coronavirus Nucleocapsid Proteins in Antiviral Drug Design. *Journal of Medicinal Chemistry* 63(6), 3131-3141. doi: 10.1021/acs.jmedchem.9b01913.
